# 

**Published:** 1906-09-08

**Authors:** 


CONTENTS.

**Education General and Medical** - 397
**The Out-Patient Problem** - 398
**Annotations** - 399
**Medical Opinion and Movement** - 400
**Hospital Clinics—**
Some Observations on Diseases of the Digestive Organs - 401
**Progress in Medicine and Surgery—**
Surgery - 403
Some Notes on Common Skin Diseases - 404
**New Appliances and Things Medical** - 406
**Special Analytical and Health Commission on Tinned and Preserved Food** - 407
**The Book World of Medicine and Science** - 409
**Hospital Administration—**
Isolation Hospital for Burslem - 410
The Relation of Insanity to Civilisation.—II. - 411
The Fitting and Furnishing of Wards and Operating Theatres 412
The Aertex Cellular Clothing Factory, Swindon - 413
The Story of the Insane from Year to Year - 414

*NURSING SECTION.*

**Notes on News from the Nursing World—**
The Value of Fire-drill—German Nurses in England—The Care of Pauper Imbeciles—The Cottage Hospital Matron—The Ignorant Midwife still Abroad—The Obligations of Guardians—The Resignations at an Irish Infirmary—Stepney and Malta—A Hint to School Nurses—The District Nurse in the Isle of Wight—A Compliment to Miss Sidney Browne—Queen Alexandra's Military Nursing Service—Progress of the Indian Nursing Association—Glasgow Lock Hospital—Entertainment to Sussex Nurses—Examination at Lambeth Infirmary—Salaries in Tasmania—Bequests to Nurses—Short Items - 323–326
**The Nursing Outlook** - 327
**Abdominal Surgery** - 328
**The Nurses' Clinic** - 329
**Incidents in a Nurse's Life** - 330
**A French War with Consumption** - 330
**The Two Matrons: an Australian Sketch** - 332
**Cottage Hospitals and Some of their Difficulties** - 333
**In a Military Hospital** - 334
**Everybody's Opinion** - 334
**Appointments** - 335
**Notes and Queries** - 336
**For Reading to the Sick** - 336

